# Lysine 2-hydroxyisobutyrylation levels determined adipogenesis and fat accumulation in adipose tissue in pigs

**DOI:** 10.1186/s40104-024-01058-9

**Published:** 2024-07-12

**Authors:** Enfa Yan, Mingyang Tan, Ning Jiao, Linjuan He, Boyang Wan, Xin Zhang, Jingdong Yin

**Affiliations:** 1 State Key Laboratory of Animal Nutrition and Feeding, College of Animal Science and Technology, China Agricultural University, Beijing, 100193 China; 2 Molecular Design Breeding Frontier Science Center of the Ministry of Education (MOE), Beijing, 100193 China; 3 College of Animal Science and Veterinary Medicine, Shandong Agricultural University, Taian, 271018 Shandong Province, China

**Keywords:** Adipogenesis, Backfat, Fat accumulation, 2-Hydroxyisobutyrylation, Intramuscular fat content, Pigs

## Abstract

**Background:**

Excessive backfat deposition lowering carcass grade is a major concern in the pig industry, especially in most breeds of obese type pigs. The mechanisms involved in adipogenesis and fat accumulation in pigs remain unclear. Lysine 2-hydroxyisobutyrylation (Khib), is a novel protein post-translational modification (PTM), which play an important role in transcription, energy metabolism and metastasis of cancer cells, but its role in adipogenesis and fat accumulation has not been shown.

**Results:**

In this study, we first analyzed the modification levels of acetylation (Kac), Khib, crotonylation (Kcr) and succinylation (Ksu) of fibro-adipogenic progenitors (FAPs), myogenic precursors (Myo) and mesenchymal stem cells (MSCs) with varied differentiation potential, and found that only Khib modification in FAPs was significantly higher than that in MSCs. Consistently, in parallel with its regulatory enzymes lysine acetyltransferase 5 (KAT5) and histone deacetylase 2 (HDAC2) protein levels, the Khib levels increased quadratically (*P* < 0.01) during adipogenic differentiation of FAPs. KAT5 knockdown in FAPs inhibited adipogenic differentiation, while HDAC2 knockdown enhanced adipogenic differentiation. We also demonstrated that Khib modification favored to adipogenic differentiation and fat accumulation by comparing Khib levels in FAPs and backfat tissues both derived from obese-type pigs (Laiwu pigs) and lean-type pigs (Duroc pigs), respectively. Accordingly, the expression patterns of KAT5 and HDAC2 matched well to the degree of backfat accumulation in obese- and lean-type pigs.

**Conclusions:**

From the perspective of protein translational modification, we are the first to reveal the role of Khib in adipogenesis and fat deposition in pigs, and provided new clues for the improvement of fat accumulation and distribution as expected via genetic selection and nutritional strategy in obese-type pigs.

**Supplementary Information:**

The online version contains supplementary material available at 10.1186/s40104-024-01058-9.

## Background

Pork is an important animal protein source in most areas of the world. Considering pigs’ feature of rapid fat accumulation, to minimize the fat deposition in subcutaneous fat and visceral fat, which are regard as unwanted fat, is a critical issue in efficient pig feeding and pork production. However, high intramuscular fat (IMF) content favors meat edible quality. In the pig industry, reducing unwanted fat accumulation while increasing IMF content is desirable, which requires a more accurate understanding on the mechanisms involved in adipogenesis and fat accumulation in backfat subcutaneous tissue and intramuscular adipocytes. The Laiwu pig is a native Chinese breed characterized by excellent meat edible quality, especially high IMF content (10.3% ± 0.19%), but with shortcomings of obese-type pigs, such as excessive backfat accumulation, slow lean growth, and low feed conversion [1]. The Duroc pig is a typical lean-type breed and usually serves as terminal sire in pork production, with the features of faster growth but relative lower IMF content (3.04% ± 0.33%) compared with obese-type pigs [2]. Therefore, how to reduce backfat deposition in obese-type pigs and increase IMF content in lean-type pigs and consequently improve meat quality attracts intensive interest from the pig industry.

An increasing number of studies were subjected to explore fat deposition and distribution in pigs from the genome, transcriptome, proteome and epigenetic modifications [3, 4]. However, the regulation of adipogenesis and subsequent fat accumulation remains unclear.

Recently, protein post-translational modification (PTM), as a new epigenetic modification has been known to confer new properties to the modified proteins, including changes in enzyme activity, subcellular localization, interaction partners, protein stability, and binding to DNA, serves an important role in regulating life activities [5]. In particular, the newly discovered lysine 2-hydroxyisobutyrylation (Khib) is an evolutionarily conserved PTM with a distinct genomic distribution and unique chemical structure [6]. The 2-hydroxyisobutyric acid, providing 2-hydroxyisobutyryl to lysine Khib by generating 2-hydroxyisobutyryl CoA, is a short-chain fatty acid produced by the metabolism of intestinal microorganisms and present at micromolar concentrations in a wide range of human biological fluids, including blood, urine, and feces [6–10]. Current studies have identified a variety of lysine acylation modification regulatory enzymes, such as histone deacetylases (HDAC1, HDAC2, and HDAC3) and the lysine acetyltransferase (e.g., KAT5, P300), regulating Khib modification of key proteins [11–15]. These enzymes could utilize co-substrates produced by cell metabolism to establish potential links between nutrition, metabolism, and gene expression [16].

Khib has been reported to regulate energy metabolism such as the citric acid cycle, fatty acid metabolism, and pyruvate metabolism [7], but it is not clear whether it regulates adipogenic differentiation and fat deposition. In this study, we explore the role of Khib in adipogenesis and fat accumulation by comparing Khib levels in the dorsal subcutaneous adipose tissue and fibro-adipogenic progenitors (FAPs) of lean- and obese-type pigs, respectively, as well as in FAPs and mesenchymal stem cells (MSCs) derived from neonatal skeletal muscle of pigs. This study would shed a highlight on the understanding of a precise regulation of adipogenesis and fat accumulation in adipose tissue and skeletal muscle in pigs, thereby providing a new strategy for pig breeding and nutritional regulation.

## Materials and methods

### Experiment animal and tissue sample collection

Neonatal (within 3 days of age, *n* = 4) and adult (*n* = 8) Laiwu and Duroc pigs were obtained from the Laiwu Pig Original Breeding Farm (Laiwu, Shandong Province, China) and Beijing Pig Breeding Center (Beijing, China), respectively. The pigs of the same breed were selected from different litters. After neonatal pigs being euthanized, 1.5 g of *Longissimus dorsi* muscles were separated for the isolation of FAPs, MSCs, and myogenic precursors (Myo).

Laiwu and Duroc pigs were raised on a corn-soybean meal-based diet formulated according to NRC (2012) [17] and Laiwu pig commercial pig feeding standards (DB 37/T 3672–2019) [18], respectively. Pigs were allowed ad libitum access to feed and clean drinking-water. When reached the marketing weight (90–100 kg for Laiwu pigs and 110–120 kg for Duroc pigs), pigs were transported to the local abattoirs, electrically stunned and killed humanely after at least 8 h rest, and were exsanguinated and eviscerated according to the standard commercial procedure. Approximately 5 g of the dorsal subcutaneous adipose tissue (backfat) was harvested at the 10–12 rib and stored at –80 °C for further analysis [19].

### Cell isolation, culture and differentiation

Skeletal muscle-derived FAPs, Myo and MSCs were isolated by the preplate technique according to previous studies [20, 21]. In a brief, about 1.5 g of the *longissimus dorsi* muscle over the last rib was manually minced. Then, muscle was digested by protease (0.17%, Sigma-Aldrich, Louis, MO, USA) for 45 min and collagenase-type XI (0.15%, Sigma-Aldrich) for 45 min at 37 °C in a thermostatic shaker (90 r/min). The suspension was filtered through 100 μm and then 40 μm cell strainers (BD Biosciences, San Jose, USA). Cell suspension was plated in a 10 mm collagen-coated dish (Sigma-Aldrich) and cultured in growth medium (DMEM; Hyclone, Logan, USA), which is containing 10% FBS (Gibco, New York, USA), 2 mmol/L glutamine (Hyclone), antibiotics (100 U/mL of penicillin and 0.1 mg/mL of streptomycin), 5 ng/mL basic fibroblast growth factor (bFGF, PeproTech, Burlington, USA). The first (0–2 h), second (2–74 h), and third (74–122 h) sets of adherent cells were FAPs, Myo and MSCs.

To induce adipogenic differentiation, cells at 80%–90% confluence were switched to differentiation medium (DM) containing 10% FBS, insulin (10 μg/mL), 0.5 mmol/L 1-methyl-3-isobutylmethylxanthine, and 1 μmol/L dexamethasone for 3 d. After that, DM was replaced with maintenance medium (DMEM containing 10% FBS and 10 μg/mL insulin) for 6 d. To induce myogenic differentiation, cells at 80%–90% confluence were treated with DM containing 2% horse serum for 6 d.

### Cell Khib proteome

Approximately 5 × 10^7^ MSCs and FAPs were harvested, respectively. The cells were sonicated in lysis buffer (8 mol/L urea, 1% protease inhibitor cocktail) on ice to extract total cellular protein. Liquid chromatography–tandem mass spectrometry (LC–MS/MS) was employed to profile cellular protein using the Tandem Mass Tag (TMT) with the assistance of PTM Biolabs Inc. (Hangzhou, Zhejiang, China). For PTM experiments, 3 μmol/L TSA and 50 mmol/L NAM were added as inhibitors of acetylation in the lysis buffer. After centrifugation at 12,000 × *g* for 10 min at 4 °C, the protein fraction in the supernatant was collected and protein concentration of samples was measured using BCA kit according to the manufacturer’s instructions.

As for trypsin digestion, the protein solution was reduced with 5 mmol/L dithiothreitol for 30 min at 56 °C and then alkylated with 11 mmol/L iodoacetamide for 15 min at room temperature in darkness. The protein sample was then diluted by adding 100 mmol/L triethylammonium bicarbonate buffer (TEAB) to a urea concentration less than 2 mol/L. Subsequently, trypsin was added at 1:50 trypsin-to-protein mass ratio for the first digestion overnight and 1:100 trypsin-to-protein mass ratio for a second 4 h-digestion. Finally, the peptides were desalted by C18 SPE column (Phenomenex, Torrance, USA).

For TMT labeling, tryptic peptides were firstly dissolved in 0.5 mol/L TEAB. Each channel of peptide was labeled with their respective TMT reagent according to the manufacturer’s protocol (ThermoFisher Scientific, Waltham, USA). Five microliters of each sample were pooled, desalted and analyzed by MS to check the labeling efficiency. Then, the pooled samples were quenched by adding 5% hydroxylamine, desalted with Strata X C18 SPE column, and dried by vacuum centrifugation.

To enrich modified peptides, tryptic peptides dissolved in nuclear and cytoplasmic extraction (NETN) buffer (100 mmol/L NaCl, 1 mmol/L EDTA, 50 mmol/L Tris–HCl, 0.5% NP-40, pH 8.0) were incubated with pre-washed antibody beads (PTM Biolabs Inc.) at 4 °C overnight with gentle shaking. Then the beads were washed four times with NETN buffer and twice with double-distilled H_2_O. The bound peptides were eluted from the beads with 0.1% trifluoroacetic acid. Finally, the eluted fractions were combined and vacuum-dried. For LC–MS/MS analysis, the resulting peptides were desalted with C18 ZipTips (Millipore) according to the manufacturer’s instructions.

As for LC–MS/MS analysis, the peptides were dissolved in 0.1% formic acid (solvent A), directly loaded onto a home-made reversed-phase analytical column (15 cm length, 75 μm i.d.). The gradient was comprised of an increase from 6% to 23% solvent B (0.1% formic acid in 98% acetonitrile) over 26 min, 23% to 35% in 8 min and climbing to 80% in 3 min, then holding at 80% for the last 3 min, all at a constant flow rate of 400 nL/min on an EASY-nLC 1000 UPLC system (ThermoFisher Scientific).

The peptides were subjected to NSI source followed by tandem mass spectrometry (MS/MS) in Q ExactiveTM Plus (Thermo) coupled online to the UPLC. The electrospray voltage applied was 2.0 kV. The m/z scan range was 350 to 1,800 for full scan, and intact peptides were detected in the Orbitrap at a resolution of 70,000. Peptides were then selected for MS/MS using NCE setting as 28 and the fragments were detected in the Orbitrap at a resolution of 17,500. A data-dependent procedure that alternated between one MS scan followed by 20 MS/MS scans with 15.0 s dynamic exclusion. Automatic gain control (AGC) was set at 5E4. Fixed first mass was set as 100 *m/z*. The resulting MS/MS data were processed using MaxQuant search engine (v.1.6.15.0). Tandem mass spectra were searched against the Sus_scrofa_9823_PR_20180816 database (40,708 entries) concatenated with reverse decoy database. Trypsin/P was specified as cleavage enzyme allowing up to two missing cleavages. The mass tolerance for precursor ions was set as 20 ppm in first search and 5 ppm in main search, and the mass tolerance for fragment ions was set as 0.02 Da. Carbamidomethyl on Cys was specified as fixed modification, and acetylation on protein N-terminal and oxidation on Met were specified as variable modifications. FDR was adjusted to < 1%.

Khib sites with fold-change (FC) > 1.20 or < 0.83 and *P* < 0.05 were considered as differentially expressed Khib sites (DEKSs). The proteins corresponding to DEKSs are differentially expressed Khib-modified proteins (DEKPs).

Bioinformatics analysis. Subcellular localization, Clusters of Orthologous Groups (COG)/EuKaryotic Orthologous Groups (KOG), Gene Ontology (GO) and Kyoto Encyclopedia of Genes and Genomes (KEGG) enrichment analysis were performed with the differentially modified proteins by the follows corresponding database or software, including Wolfpsort (subcellular localization), COG/KOG database (COG/KOG), UniProt-GOA database and InterProScan software (GO), and KEGG database, KEGG mapper and KAAS software (KEGG). Significantly enriched GO terms or KEGG pathways were identified by Fisher exact test, and *P* < 0.05 was considered as the threshold. Gene set enrichment analysis (GSEA) of the regulated pathways with the GSEA v4.0.3 software and KEGG database.

### siRNA transfection

Cells were transfected with siRNA using Lipofectamine 3000 Transfection Reagent (Invitrogen, Waltham, USA) when cells were grown to 80%–90% fusion [21]. siRNA information was provided in Table S1. After transfection for 48 h, cells were collected or subjected to adipogenic induction.

### Western blotting

Protein samples were extracted from cells and the dorsal subcutaneous fat tissue using RIPA lysis buffer (containing protease inhibitors and protein phosphatase inhibitors; Huaxingbio, Beijing, China) and quantified using the BCA protein assay kit (ThermoFisher). The 30–50 µg of protein samples were separated by 10% SDS-PAGE and transferred to ImmunBlot™ polyvinylidene fluoride (PVDF) membranes (Merck Millipore, Darmstadt, Germany) using the BIO-RAD mini protein tetra system. The PVDF membranes were blocked with 5% bovine serum albumin (BSA) solution and then incubated with primary antibodies overnight at 4 °C. Then, the PVDF membranes were washed twice in TBST and incubated for 60 min at room temperature and in the dark condition using the corresponding secondary antibodies. Exposure and imaging were performed using an Odyssey Clx fluorescence imaging system (LI-COR Biotechnology, Lincoln, USA). Gray analysis of blots was performed using ImageJ (National Institutes of Health, Bethesda, USA) [19]. β-Actin or β-tubulin was used as the internal control. The antibody information and the dilution ratios of primary to secondary antibodies were shown in Table S2.

### RNA extraction and qRT-PCR

Total RNA from cells or backfat samples was extracted using RNAiso (Takara Bio Inc., Tokyo, Japan) and then reversed to cDNA using a reverse transcription kit (Takara). Relative expression of genes was quantified by qRT-PCR. The mRNA relative expression levels of target genes were calculated by the 2^−ΔΔCt^ method using *GAPDH* as an internal control [22]. Based on the mRNA sequences of pigs, primers were designed using Primer premier 6.0 software (see Table S3 for primer information) and synthesized by (GENEWIZ Biotechnology Co., Ltd., Jangsu, China).

### Immunofluorescence

After adipogenic or myogenic differentiation, cells were fixed with 4% paraformaldehyde and permeabilized with 0.5% TritonX-100 for 10 min. Cells were then blocked with 5% BSA for 60 min at room temperature and incubated with primary antibodies against perilipin 1 (PLIN1) and Myosin (Sigma, dilution ratio, 1:200) overnight at 4 °C. After washed with PBS, cells were incubated with secondary antibodies (antibody to Rabbit IgG-Alexa Fluor 594, Abcam, dilution ratio, 1:1,000) for 60 min at room temperature. Finally, nuclei were stained with DAPI (1 µg/mL) for 10 min [21]. Staining was observed and photographed using an inverted fluorescence microscope (Olympus, Tokyo, Japan). The information of antibodies was shown in Table S2.

### Biochemical component concentrations and fatty acid composition of backfat

About 100 mg of backfat samples was homogenized and the contents of protein (BCA protein assay kit), triglyceride (Angle Gene, Nanjing, China) and cholesterol (Angle Gene) were determined using commercial kits according to the manufacturer’s instructions.

To determine the fatty acid composition of backfat samples, lyophilized backfat (150 mg) were mixed with acetyl/anhydrous methanol (4 mL; 1/10, v/v), n-hexane (1 mL), and internal standard fatty acid solution (1 mL; 1 mg/mL C11:0). The mixture was kept in a water bath at 80 °C for 2.5 h. After cooling to room temperature, the mixture was combined with K_2_CO_3_ (5 mL; 70 g/L) and centrifuged at 800 × *g* for 3 min. The supernatant was analyzed by gas chromatography (Agilent Technologies Inc., Santa Clara, CA, USA), and the fatty acid content is calculated by the following formula: *X*_*i*_ = (*A*_si_ × *m*_stdi_ × *F*_*j*_)/(*A*_stdi_ × *m*) × 100. *X*_*i*_, the content of each fatty acid in the sample (mg/100 g). *A*_si_, peak area of each fatty acid in the sample determination solution. *m*_stdi_, mass of the standard contained in the standard working solution of fatty acid triglycerides drawn up in the preparation of the standard assay solution, unit is mg. *F*_*j*_, conversion factors of each fatty acid triglyceride into fatty acid. *A*_stdi_, standard determination of the peak area of each fatty acid in the liquid. *m*, weighing mass of the sample, unit is gram (g). Fatty acid composition was presented as mg/g of backfat basis [23].

### Measurement of enzyme activities

Activities of acetyl-CoA carboxylase (ACC), adipose triglyceride lipase (ATGL), fatty acid synthase (FASN), hormone-sensitive triglyceride lipase (HSL) and stearoyl-CoA desaturase (SCD) were determined using commercial kits (Angle Gene, Nanjing, China) according to the manufacturer’s instructions.

### Statistical analysis

Data were expressed as mean ± SEM. Statistical significance was assessed by linear and quadratic regression analysis or Student’s *t*-test (two-tailed) using SAS (Version 9.4, SAS Institute, Cary, NC, USA). A value of *P* < 0.05 was considered significant, and 0.05 < *P* < 0.10 was considered to have a trend.

## Results

### Backfat composition and lipid metabolism in different types of pigs

As shown in Fig. S1, at marketing weight, the contents of protein (*P* < 0.001, Fig. S1A) and total cholesterol (TC) (*P* = 0.013, Fig. S1B) and triglyceride (TG) (*P* = 0.006, Fig. S1C) of the backfat were significantly lower in obese-type pigs than those in lean-type pigs while the contents of total fatty acid (*P* < 0.001, Fig. S1D) were significantly higher in obese-type pigs. Moreover, the concentrations of C10:0, C12:0, C14:0, C16:0, C16:1, C18:0, C18:1n9c, C20:1, C22:0, C22:1n9, C22:2 and C23:0 in the backfat were significantly higher in obese-type pigs compared with those in lean-type pigs (*P* < 0.05, Fig. S2A), while the concentrations of C15:0, C17:0, C18:2n6c, C18:3n3, C20:4n6 and C20:3n3 were lower in obese-type pigs (*P* < 0.05, Fig. S2A). Furthermore, the concentrations of saturated fatty acid (SFA) and monounsaturated fatty acid (MUFA) and the ratio of n-6/n-3 polyunsaturated fatty acids (PUFA) were higher in the backfat of obese-type pigs than those of lean-type pigs (*P* < 0.01, Fig. S2B and C). The concentrations of n-3 and n-6 PUFA (Fig. S2B) and the ratio of PUFA/SFA (*P* < 0.01, Fig. S2D) in obese-type pigs were lower than lean-type pigs. We also analyzed the activities of enzymes involved in fatty acid mobilization, include ATGL and HSL, as well as those of enzymes involved in fatty acid synthesis, including ACC, FASN and SCD. The activity of ATGL, a key enzyme for the release of fatty acids from TG during lipolysis, was lower in the backfat of obese-type pigs (*P* = 0.001, Fig. S3A), while no significant change was observed in the activity of HSL between the two types of pigs. The activities of key enzymes for fatty acid synthesis, including ACC and FASN were also lower in obese-type pigs (*P* < 0.01, Fig. S3C and D). Meanwhile, the activity of SCD, the rate-limiting enzyme for MUFA synthesis, was higher in the backfat of obese-type pigs (*P* = 0.006, Fig. S3E).

### Khib modification levels of FAPs, Myo and MSCs

**Fig. 1:**
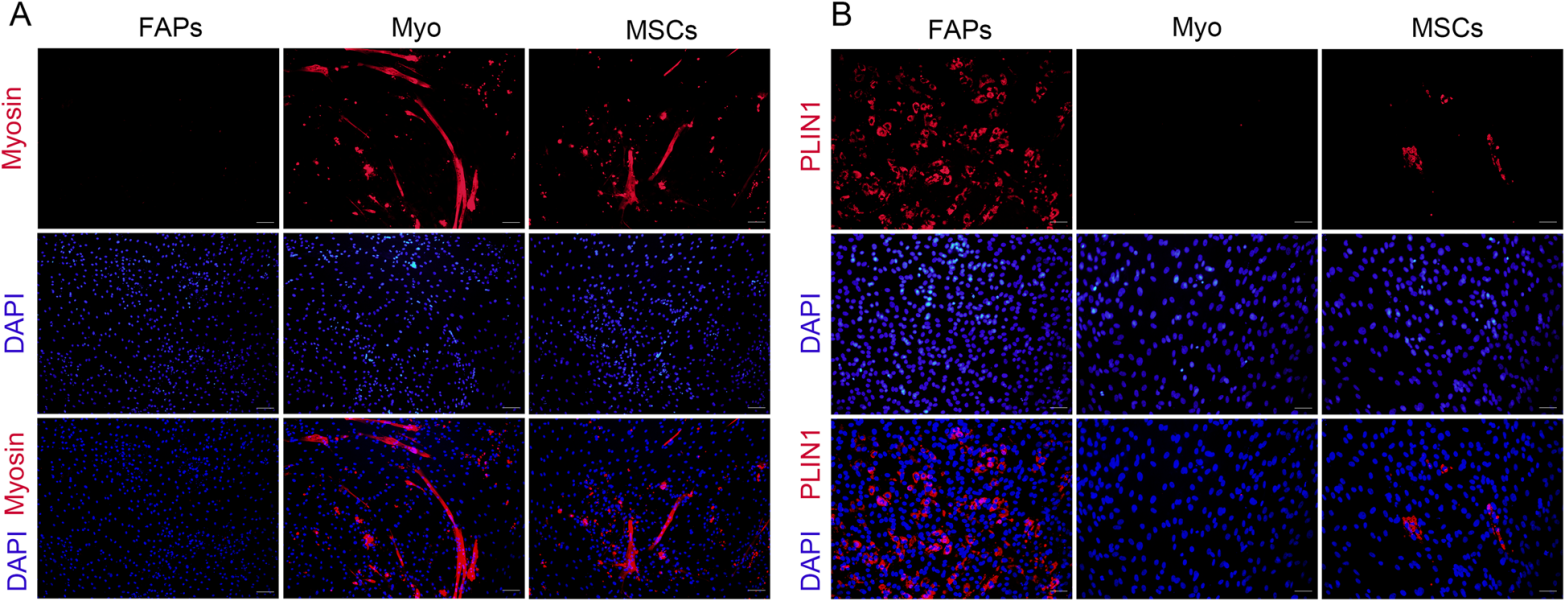
Comparison of cell differentiation ability between FAPs, Myo and MSCs. **A** Immunostaining with myosin antibody of FAPs, Myo and MSCs after myogenic induction for 6 d, with a scale of 100 μm (*n* = 4). **B** Immunostaining with PLIN1 antibody of FAPs, Myo and MSCs after adipogenic induction for 9 d, with a scale of 50 μm (*n* = 4)

In this study, FAPs, Myo and MSCs derived from the skeletal muscle of neonatal pigs were employed to investigate the role of protein PTM in adipogenesis. First, the divergent differentiation potential of these three cell populations was shown in Fig. 1. Immunofluorescence staining showed that Myo and MSCs could differentiate into myotubes, while FAPs had no myogenic differentiation capacity (Fig. 1A). In contrast, accumulating lipids were observed in FAPs and MSCs after adipogenic differentiation, but more mature adipocytes were observed in FAPs than in MSCs. However, Myo had no adipogenic differentiation capacity (Fig. 1B).

**Fig. 2:**
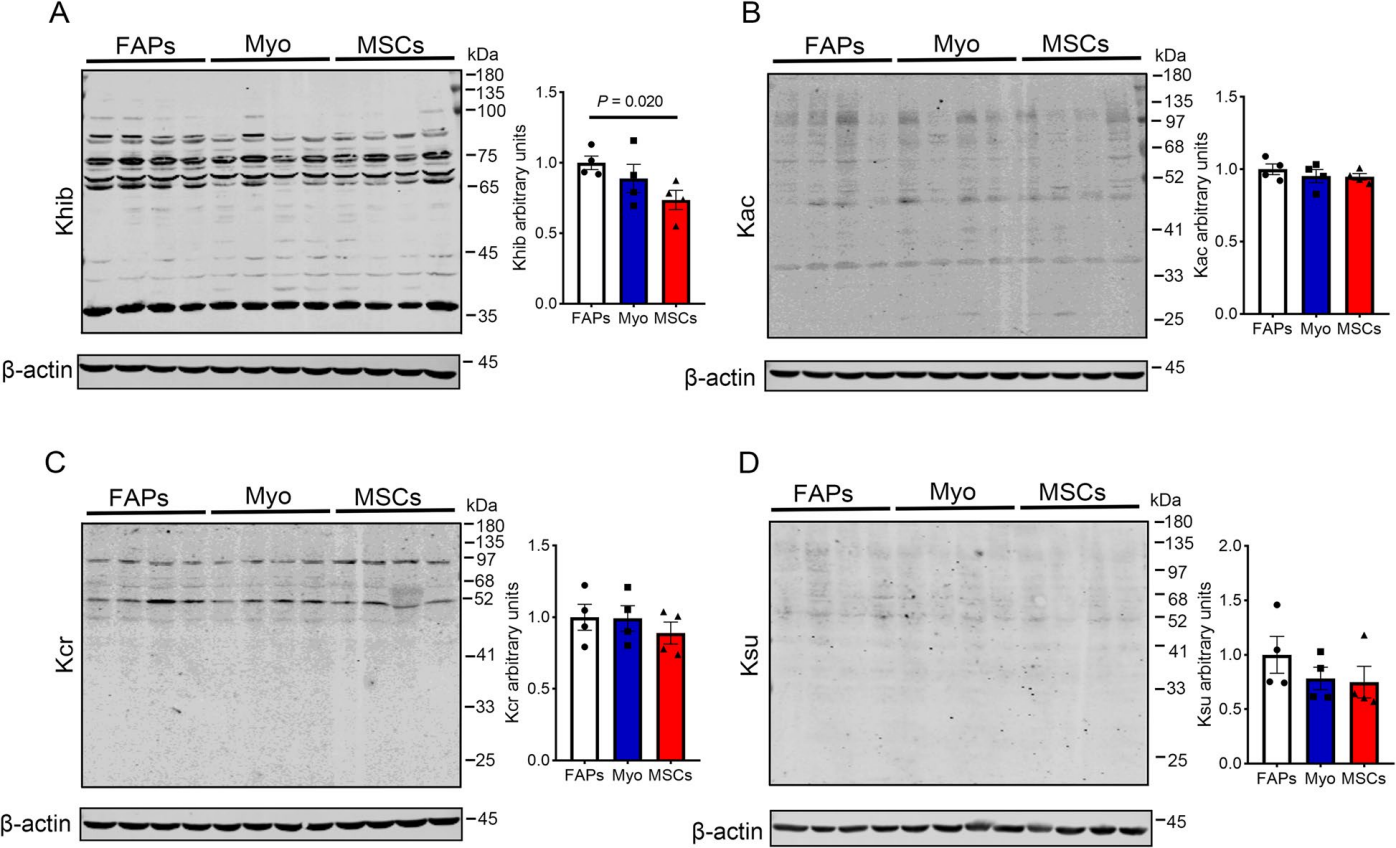
Khib, Kac, Kcr and Ksu modification levels in FAPs, Myo and MSCs. **A** The level of Khib modification in FAPs, Myo and MSCs. **B** The Kac modification levels of FAPs, Myo and MSCs. **C** The Kcr modification level of FAPs, Myo and MSCs. **D** The Ksu modification level of FAPs, Myo and MSCs. The data are presented as the mean ± SEM, *n* = 4

**Fig. 3:**
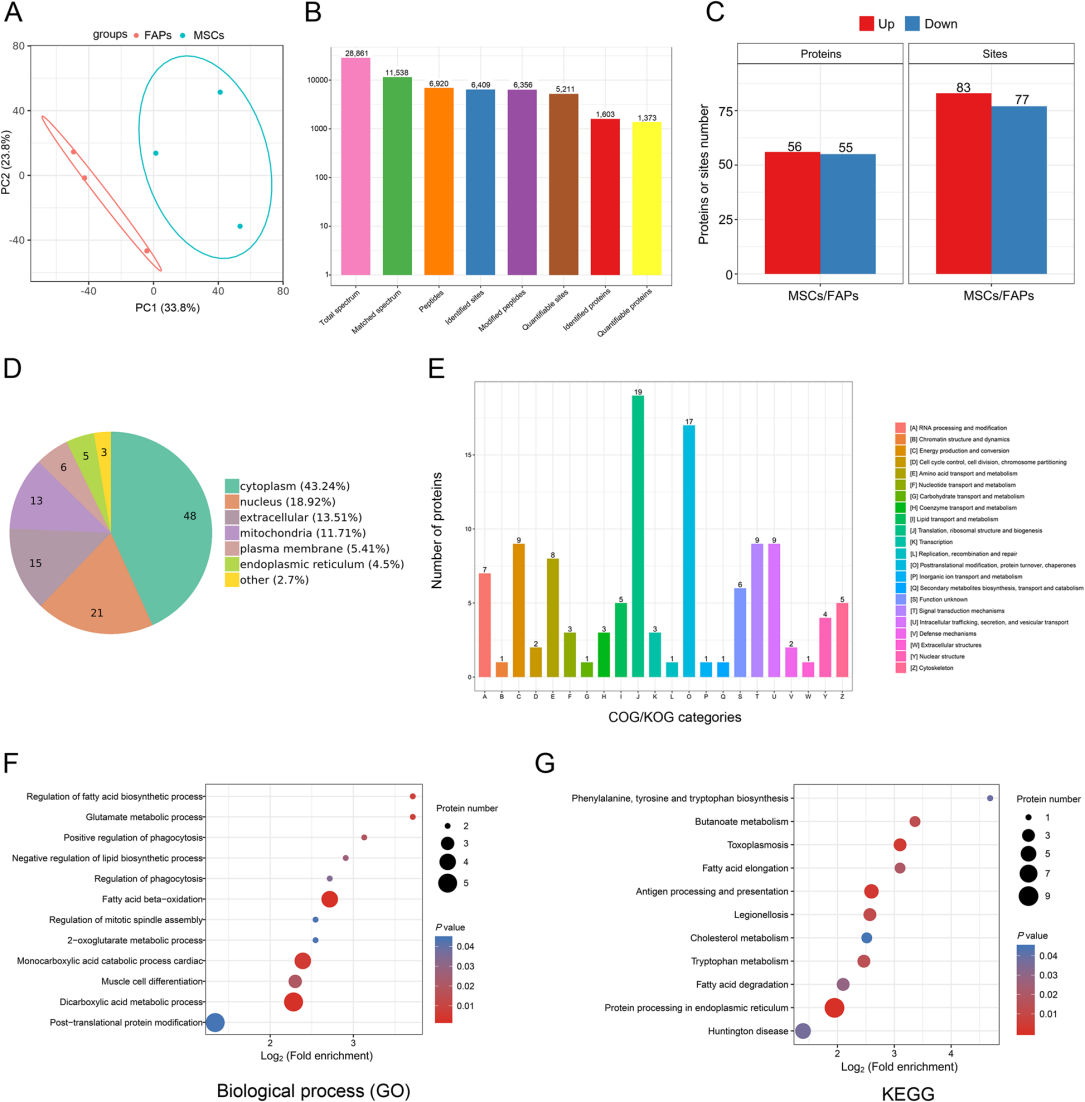
Comprehensive landscape of Khib in MSCs and FAPs. **A** PCA plot showing differences in the characterization of FAPs and MSCs. **B** Overview of protein identification. **C** The subcellular distribution of Khib modified proteins. **D** The statistical analysis of Khib modified proteins and modification sites of FAPs and MSCs. **E** COG/KOG enrichment analysis of Khib modified proteins. **F** Part of enriched biological process. **G** Part of enriched KEGG. Contrast strategy: MSCs vs. FAPs, *n* = 3

**Fig.4:**
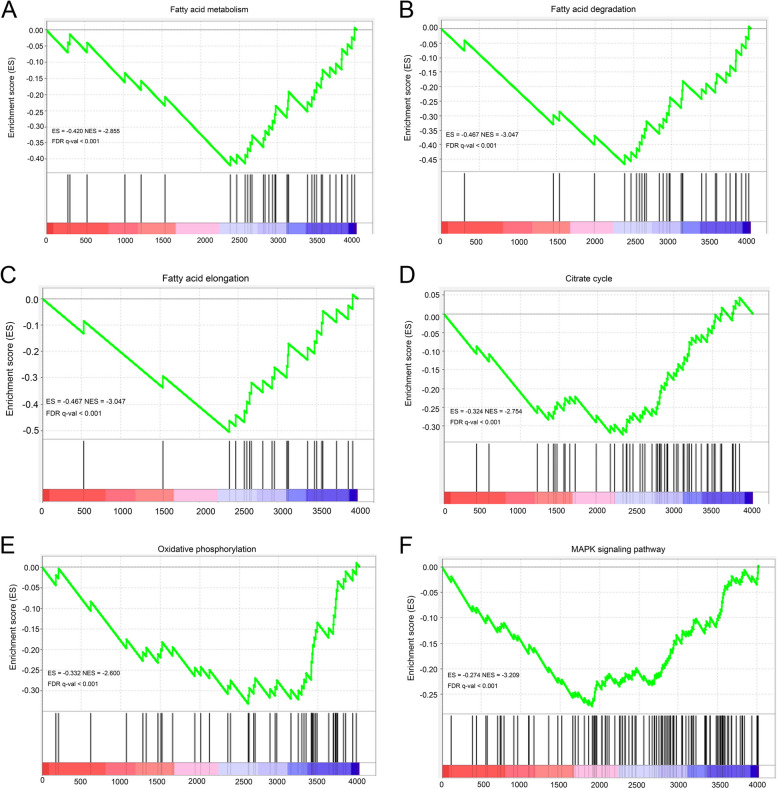
Lipid and energy metabolism pathways enriched by Khib modified proteins are down-regulated in MSCs. **A–D** GSEA results for KEGG pathway enrichment comparing FAPs and MSCs using Khib modified protein dataset. **A** Fatty acid metabolism. **B** Fatty acid degradation. **C** Fatty acid elongation. **D** Citrate cycle. **E** Oxidative phosphorylation. **F** MAPK signaling pathway. Contrast strategy: MSCs vs. FAPs, *n* = 3

Protein PTM patterns were next determined using a panel of pan antibodies. The Khib level of FAPs was higher than that of MSCs (*P* = 0.020, Fig. 2A), and there was no difference in levels of acetylation (Kac), crotonylation (Kcr) and succinylation (Ksu) among FAPs, Myo and MSCs (Fig. 2B–D). Therefore, we further analyzed the profiling of Khib modified proteome in FAPs and MSCs. PCA analysis showed the clear separation of Lys-Khib modifications between FAPs and MSCs (Fig. 3A). A total of 28,861 secondary spectral peak patterns were detected by LC–MS/MS, and 1,373 quantifiable proteins and 5,211 quantifiable sites were identified (Fig. 3B). Using the proteomics data to correct the protein modification omics data, a total of 111 differentially expressed Khib-modified proteins (DEKPs) and 160 differentially expressed Khib sites (DEKSs) were identified. Based on the criteria of fold change FC > 1.2 or < 0.83 and *P* < 0.05, 56 DEKPs and 83 DEKSs were up-regulated in MSCs, while 55 DEKPs and 77 DEKSs were down-regulated (Fig. 3C). Bioinformatic analysis of subcellular localization showed that 48 DEKPs were located in the cytoplasm, accounting for 43.24% DEKPs, and followed by 21 DEKPs (18.92%) in cell nucleus, 15 DEKPs (13.51%) in the extracellular and 13 DEKPs (11.71%) in the mitochondria, etc. (Fig. 3D). COG/KOG enrichment analyses indicated that Khib might be involved in the functions including translation/ ribosomal structure/biogenesis, post-tranlational modification/protein turnover/chaperone, RNA processing and modification, energy production and conversion, amino acid transport and metabolism signal transduction mechanisms, intracellular trafficking/secretion/vesicular transport, lipid transport and metabolism, etc. (Fig. 3E). Further GO biological process and KEGG enrichment analysis revealed that Khib modifications were implicated with fatty acid metabolism, such as fatty acid biosynthetic process, fatty acid beta-oxidation, fatty acid elongation and fatty acid degradation were all significantly enriched (Fig. 3F and G). As shown in Table S4, GSEA results showed that 14 pathways were significantly up-regulated while 30 pathways were significantly down-regulated (FDR < 0.05, FWER *P* < 0.05). Importantly, lipid and energy metabolism pathway, including fatty acid metabolism, fatty acid degradation, fatty acid elongation, citrate cycle, oxidative phosphorylation and the MAPK signaling pathway were significantly down-regulated in MSCs (Fig. 4A–D).

### “Writer” KAT5 and “Eraser” HDAC2 of Khib affect adipogenesis in FAPs cells

**Fig. 5:**
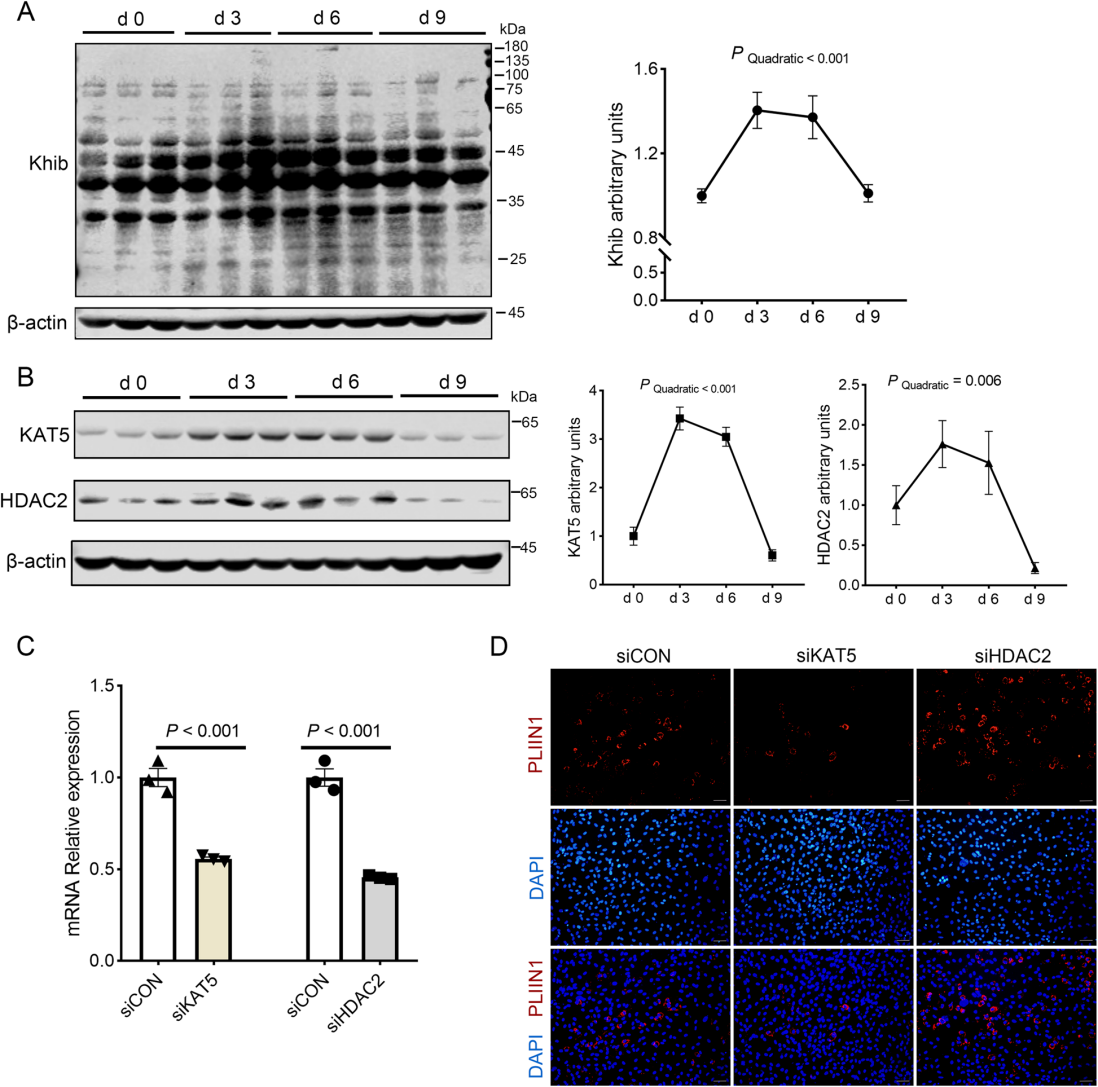
KAT5 and HDAC2 affect FAPs adipogenic differentiation. **A** The Khib modification level of FAPs after adipogenic induction for 0, 3, 6, 9 d (*n* = 3). **B** The changes of KAT5, HDAC2 protein expression in FAPs after adipogenic induction for 0, 3, 6, 9 d (*n* = 3). **C** *KAT5* and *HDAC2* knockdown efficiency (*n* = 3). **D** The effect of KAT5 and HDAC2 knockout on the expression of PLIN1 in FAPs after adipogenic induction for 3 d (*n* = 4), with a scale of 50 μm. The data are presented as the mean ± SEM

The levels of Khib were significantly increased during adipogenic differentiation (*P*_q__uadratic_ < 0.001, Fig. 5A). Importantly, “Writer” KAT5 and “Eraser” HDAC2 likewise exhibited elevated expression levels during adipogenic differentiation (*P*_quadratic_ < 0.01, Fig. 5B). To further explore the role of KAT5 and HDAC2 in adipogenic differentiation, siRNA inhibition of KAT5 and HDAC2 was successfully developed in FAPs (Fig. 5C). After 3-d of adipogenic differentiation, lipid accumulation was significantly blunted by KAT5 knockdown but enhanced by HDAC2 knockdown (Fig. 5D). Thus, Khib may be required for adipogenic differentiation.

### Khib levels in the FAPs and backfat of obese-type and lean-type pigs

**Fig. 6:**
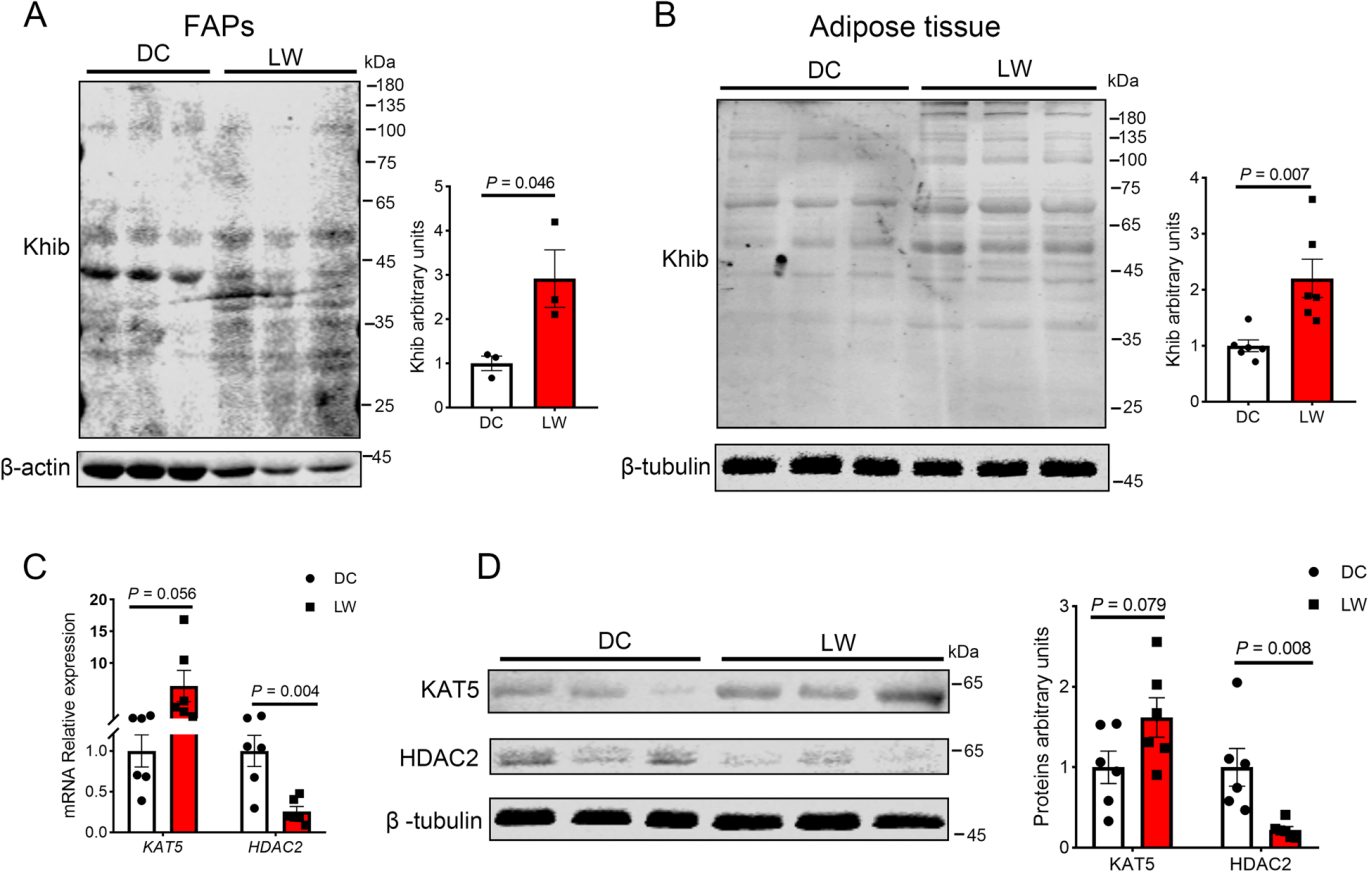
Differences in FAPs and adipose tissue Khib modification levels of obese- and lean-type pigs. **A** Khib levels in FAPs (adipogenic differentiation for 3 d) of Duroc and Laiwu pig (*n* = 3). **B** Khib levels in adipose tissue of Duroc and Laiwu pig (*n* = 6). **C** *KAT5* and *HDAC2* gene expression levels in backfat of Duroc and Laiwu pigs (*n* = 6). **D** KAT5 and HDAC2 protein expression levels in backfat of Duroc and Laiwu pigs (*n* = 6). DC, Duroc pig (lean-type pig); LW, Laiwu pig (obese-type pig). The data are presented as the mean ± SEM

After 3-d of adipogenic differentiation of FAPs from newborn obese- and lean-type pigs, cellular Khib levels were significantly higher in obese-type than in lean-type pigs (*P* = 0.046, Fig. 6A). In obese-type pigs, the Khib level of the dorsal subcutaneous adipose tissue was also higher than that in lean-type pigs (*P* = 0.007, Fig. 6B). Consistently, the mRNA and protein levels of KAT5 tended to be higher in the dorsal subcutaneous adipose tissue of obese-type pigs relative to lean-type pigs (0.05 < *P* < 0.10, Fig. 6C–D). Whereas, a significant decrease in HDAC2 levels was observed in obese-type pigs compared with lean-type pigs (*P* < 0.01, Fig. 6C–D).

## Discussion

A large number of studies have been conducted to decipher mechanisms regulating adipogenesis and fat deposition. Numerous functional genes, key regulatory loci, and changes of chromatin structure have been identified to regulate adipogenic differentiation and fat accumulation [21, 24, 25]. However, some basic issues still need to be further elucidated, such as the expression effect of functional genes varied greatly among different animal populations and the influence of environmental factors on traits. Non-histone lysine acylation modifications have recently received increasing attention for their critical roles in cell survival, gene transcription, signaling transport and cellular metabolism, as well as their susceptible to multiple environmental factors [26, 27]. In this study, the role of Khib modification in adipogenic differentiation and fat accumulation was explored by employing lean- and obese-type pigs, as well as cell populations with divergent differentiation potentials.

It has been well known that obese- and lean-type pigs have different fat-accumulation capacities. Here, we also showed that the contents of total protein, TC and TG in backfat were lower in obese-type pigs than those in lean-type pigs. Similarly, the total fatty acid content in backfat was higher in obese-type pigs, which may be the reason that the fat of obese-type pigs is more beneficial to human health relative to lean-type pigs in previously studies [28, 29]. In addition, the difference in backfat fatty acid composition was in agreement with the previous study [30]. It is generally known that fatty acid composition in pig tissues is greatly influenced by dietary fatty acid composition, and PUFA in porcine tissue are mainly derived from the absorbed dietary PUFA [31, 32]. Obese-type pigs have stronger de novo fatty acid synthesis capacity relative to lean-type pigs, resulting in their higher SFA concentration and lower PUFA concentration in backfat [29].

ATGL and HSL are the main enzymes that catalyze lipolysis and fat mobilization [33]. ATGL catalyzes lipolysis in both basal and stress states and initiates the process of hydrolysis of triglyceride (TAG) into diacylglycerol (DAG) and FFA [34]. HSL acts under the action of hormones promoting fat mobilization, and the main substrate of the action is diglycerides. Accordingly, ATGL has a greater fat mobilizing capacity than HSL [35, 36]. In this study, we found that the activity of ATGL in the dorsal subcutaneous adipose tissue of lean-type pigs was significantly higher than that of obese-type pigs, while there was no significant difference in HSL activity. It indicated that lipid mobilization in the backfat of the lean-type pig was stronger than that of obese-type pigs.

FASN and SCD are key enzymes that catalyze fatty acid synthesis in vivo. FASN undergoes a series of decarboxylation condensation reactions with malonyl-CoA to produce palmitate (C16:0) [37]. Palmitate is lengthened and/or converted to longer fatty acid chains by fatty acid elongases (ELOVs) and desaturases (SCD). Activities of ACC and FASN in the backfat of obese-type pigs were significantly lower than those in lean-type pigs, while SCD activity was higher in obese-type pigs compared with lean-type pigs, which may be attributed to the fact that obese-type pigs have reached a plateau phase of adipose tissue maturation at the marketing stage, while lean-type pigs with marketing body weight did not yet reach adipose maturity. Furthermore, it also suggested that, relative to fatty acid de novo synthesis process, fatty acid elongation catalyzed by SCD might dominate fat accumulation in the dorsal subcutaneous adipose tissue in finishing pigs.

The process of adipose tissue development involves adipogenic differentiation and subsequent fat accumulation in mature adipocytes [38]. It is generally accepted that adipocytes differentiate from mesodermal MSCs, and MSCs are oriented into FAPs and then differentiate into adipocytes [20]. Epigenetic modifications may be a key mechanism through which cells with the same set of genomes have different fates [39, 40], and the epigenetic landscape of cells implicates their past and current developmental status and may predict their future potential [40]. In this study, we comparatively analyzed the levels of Kac, Khib, Kcr and Ksu in FAPs, Myo and MSCs derived from the neonatal skeletal muscle of pigs, and found that only Khib level was higher in FAPs than MSCs. In further studies, we also found that the level of Khib was elevated during adipogenic differentiation of FAPs, and reached the highest level after 3-d of adipogenic differentiation. Meanwhile, lipid metabolism-related pathways were down-regulated in MSCs, suggesting that Khib may play a key role in the regulation of adipogenic differentiation and subsequent intracellular lipid metabolism and adipocyte maturity. Consistently, the Khib level of differentiating FAPs from obese-type pigs were higher than that from lean-type pigs.

Lysine acylation modification is catalyzed by its “Writer” (modification) and “Eraser” (de-modification) and is influenced by the substrate contents [14]. As important members of the acetyltransferases, KAT5 has been identified as a “Writer” capable of catalyzing Khib in mammalian cells in vitro and in vivo; while histone deacetylase family, such as HDAC2 and HDAC3, can act as "Erasers" to remove Khib [14]. A previous study showed that KAT5 knockout significantly reduced the amount of body fat in mice, and the inhibition of the KAT5 activity blocked TG synthesis [41]. Besides, it has been shown that HDAC2 affected fat accumulation, and a negative correlation existed between HDAC2 and human obesity index [42]. KAT5 and HDAC2 are known key enzymes that regulate Khib modification of histone [7], but their regulation of non-histone Khib has not been reported. In this study, we found that the protein levels of HDAC2 and KAT5 exhibited a significant quadratic increase during adipogenic differentiation, consistent with the variations in Khib levels, which suggested that KAT5 and HDAC2 may regulate adipogenic differentiation and fat accumulation by regulating Khib. Feng et al. [43] revealed that elevated HDAC2 activity attenuates hepatic ectopic fat accumulation in MKP-3 (an enzyme that inhibits HDAC2 activity) knockout mice fed with a high-fat diet, and in vitro studies also demonstrated that the inhibition of HDAC2 expression in hepatocytes elevated the expression of adipose-specific genes. In the present study, knockdown of KAT5 resulted in a decrease in the lipid accumulation of FAPs, whereas HDAC2 knockdown enhanced the adipogenic differentiation capacity of FAPs. Compared with lean-type pigs, HDAC2 expression levels were also lower in the backfat of obese-type pigs, but KAT5 expression levels tended to be higher in obese-type pigs. In this regard, we speculate that KAT5 and HDAC2 may regulate adipogenic differentiation and fat accumulation in pigs by regulating Khib levels in FAPs.

For further validation, we tested the Khib level in the backfat of lean- and obese-type pigs, and demonstrated that the Khib level in the backfat was higher in obese-type pigs than that in lean-type pigs. These results suggested that Khib may play an important role in the regulation of adipogenesis and fat accumulation capacity.

## Conclusions

We are first to reveal that Khib may play an important role in the regulation of adipogenesis and fat accumulation in pigs at protein PTM level, and Khib level favor adipogenesis and fat accumulation capacity. It can also provide a new clue to regulate fat accumulation and distribution in pigs via genetic selection or nutritional regulation.

### Supplementary Information

**Additional file 1**. **Fig. S1** Comparison of TC, TG, total fatty acid and protein contents of backfat between obese- and lean-type pigs. TC, total cholesterol. TG, Triglyceride. DC, Duroc pig (lean-type pig). LW, Laiwu pig (obese-type pig). The data are presented as the mean ± SEM, *n* = 6, 7, or 8.

**Additional file 2**. **Fig. S2** Comparison of fatty acid composition of backfat between obese- and lean-type pigs. **A** and **B** Fatty acid composition of backfat in obese- and lean-type pigs (*n* = 6, 7 or 8). **C** The ratio of n-6/n-3 polyunsaturated fatty acids (PUFA) of backfat in obese- and lean-type pigs (*n* = 6, 7 or 8). **D** The PUFA/SFA ratio of backfat in obese- and lean-type pigs (*n* = 6, 7, or 8). DC, Duroc pig (lean-type pig). LW, Laiwu pig (obese-type pig). The data are presented as the mean ± SEM.

**Additional file 3**. **Fig. S3** Comparison of activities of enzymes related to fatty acid metabolism of backfat between obese- and lean-type pigs. DC, Duroc pig (lean-type pig). LW, Laiwu pig (obese-type pig). The data are presented as the mean ± SEM, *n* = 6, 7 or 8.

**Additional file 4**. **Table S1** Sequences of siRNA. **Table S2** Antibodies information. **Table S3** Primer sequences of target genes used for qRT-PCR assays. **Table S4** Differential regulatory pathways of Khib modified proteins of FAPs and MSCs in GSEA. 

## Data Availability

The data analyzed during the current study are available from the corresponding author on reasonable request.

## Abbreviations

C10:0: Capric acid
C12:0: Lauric acid
C14:0: Myristic acid
C15:0: Pentadecanoic acid
C16:0: Palmitic acid
C16:1: Palmitoleic acid
C17:0: Heptadecanoic acid
C18:0: Stearic acid
C18:1n9c: Methyl oleate
C18:2n6c: Methyl (Z, Z)-9,12-octadecadienoate
C18:3n3: Alpha-linolenic acid
C20:1: Eicosenoic acid
C20:3n3: Eicosatrienoic acid
C20:4n6: Arachidonic acid
C22:0: Behenic acid
C22:1n9: Methyl *cis*-9-docosenoate
C22:2: *cis*-13,16-Docosadienoic acid
C23:0: Methyltricosanoate
DC: Duroc pig
ELOVs: Fatty acid elongases
FADS: Fatty acid desaturatase
FAPs: Fibro-adipogenic progenitor
HDAC2: Histone deacetylase 2
IMF: Intramuscular fat
Kac: Acetylation
KAT5: Lysine acetyltransferase 5
Kcr: Crotonylation
Khib: 2-hydroxyisobutyrylation
Ksu: Succinylation
LW: Laiwu pig
MSCs: Mesenchymal stem cells
MUFA: Monounsaturated fatty acid
Myo: Myogenic precursors
NETN: Nuclear and cytoplasmic extraction
PLIN1: Perilipin 1
PTM: Protein post-translational modification
PUFA: Polyunsaturated fatty acids
SFA: Saturated fatty acid
TC: Total cholesterol
TEAB: Triethylammonium bicarbonate buffer
TG: Triglyceride

## References

[1] Wang Y, Zhang H, Yan E, He L, Guo J, Zhang X (2023). Carcass and meat quality traits and their relationships in Duroc × Landrace × Yorkshire barrows slaughtered at various seasons. Meat Sci.

[2] Li M, Tian S, Jin L, Zhou G, Li Y, Zhang Y (2013). Genomic analyses identify distinct patterns of selection in domesticated pigs and Tibetan wild boars. Nat Genet.

[3] Wang L, He T, Zhang X, Wang Y, Qiu K, Jiao N (2021). Global transcriptomic analysis reveals Lnc-ADAMTS9 exerting an essential role in myogenesis through modulating the ERK signaling pathway. J Anim Sci Biotechno.

[4] Sun WJ, He T, Qin CF, Qiu K, Zhang X, Luo YH (2017). A potential regulatory network underlying distinct fate commitment of myogenic and adipogenic cells in skeletal muscle. Sci Rep.

[5] Verdin E, Ott M (2015). 50 years of protein acetylation: from gene regulation to epigenetics, metabolism and beyond. Nat Rev Mol Cell Biol.

[6] Dai L, Peng C, Montellier E, Lu Z, Chen Y, Ishii H (2014). Lysine 2-hydroxyisobutyrylation is a widely distributed active histone mark. Nat Chem Biol.

[7] Huang H, Luo Z, Qi S, Huang J, Xu P, Wang X (2018). Landscape of the regulatory elements for lysine 2-hydroxyisobutyrylation pathway. Cell Res.

[8] Park J, Chen Y, Tishkoff DX, Peng C, Tan M, Dai L (2013). SIRT5-mediated lysine desuccinylation impacts diverse metabolic pathways. Mol Cell.

[9] Nishida Y, Rardin MJ, Carrico C, He W, Sahu AK, Gut P (2015). SIRT5 regulates both cytosolic and mitochondrial protein malonylation with glycolysis as a major target. Mol Cell.

[10] Rardin MJ, He W, Nishida Y, Newman JC, Carrico C, Danielson SR (2013). SIRT5 regulates the mitochondrial lysine succinylome and metabolic networks. Cell Metab.

[11] Bowers EM, Yan G, Mukherjee C, Orry A, Wang L, Holbert MA (2010). Virtual ligand screening of the p300/CBP histone acetyltransferase: identification of a selective small molecule inhibitor. Chem Biol.

[12] Sabari BR, Tang Z, Huang H, Yong-Gonzalez V, Molina H, Kong HE (2015). Intracellular crotonyl-CoA stimulates transcription through p300-catalyzed histone crotonylation. Mol Cell.

[13] Wei W, Liu X, Chen J, Gao S, Lu L, Zhang H (2017). Class I histone deacetylases are major histone decrotonylases: evidence for critical and broad function of histone crotonylation in transcription. Cell Res.

[14] Huang H, Tang S, Ji M, Tang Z, Shimada M, Liu X (2018). P300-mediated lysine 2-hydroxyisobutyrylation regulates glycolysis. Mol Cell.

[15] Madsen AS, Olsen CA (2012). Profiling of substrates for zinc-dependent lysine deacylase enzymes: HDAC3 exhibits decrotonylase activity in vitro. Angew Chem Int Ed Engl.

[16] Kaelin WG, McKnight SL (2013). Influence of metabolism on epigenetics and disease. Cell.

[17] NRC (National Research Council). Nutrient requirements of swine. Eleventh Revised Edition. Washington: National Academic Press; 2012.

[18] Technical Committee on Animal Husbandry of Standardization Administration of Shandong Province, China. DB 37/T 3672—2019 Laiwu pig commercial pig feeding standard. Beijing: China Agriculture Press; 2019.

[19] Yan EF, Wang YB, He LJ, Guo JX, Zhang X, Yin JD (2022). Effects of dietary L-malic acid supplementation on meat quality, antioxidant capacity and muscle fiber characteristics of finishing pigs. Foods.

[20] Gharaibeh B, Lu A, Tebbets J, Zheng B, Feduska J, Crisan M (2008). Isolation of a slowly adhering cell fraction containing stem cells from murine skeletal muscle by the preplate technique. Nat Protoc.

[21] Xu DD, Wan BY, Qiu K, Wang YB, Zhang X, Jiao N (2023). Single-cell RNA-sequencing provides insight into skeletal muscle evolution during the selection of muscle characteristics. Adv Sci.

[22] Livak KJ, Schmittgen TD (2001). Analysis of relative gene expression data using real-time quantitative PCR and the 2^−ΔΔCT^ method. Methods.

[23] Guo JX, Yan EF, He LJ, Wang YB, Xiang YF, Zhang PG (2023). Dietary supplementation with lauric acid improves aerobic endurance in sedentary mice via enhancing fat mobilization and glyconeogenesis. J Nutr.

[24] Zhang X, Wang L, Wang Y, He L, Xu D, Yan E (2023). Lack of adipocyte IP3R1 reduces diet-induced obesity and greatly improves whole-body glucose homeostasis. Cell Death Discov.

[25] Li X, Zeng S, Chen L, Zhang Y, Li X, Zhang B (2024). An intronic enhancer of Cebpa regulates adipocyte differentiation and adipose tissue development via long-range loop formation. Cell Prolif.

[26] Narita T, Weinert BT, Choudhary C (2019). Functions and mechanisms of non-histone protein acetylation. Nat Rev Mol Cell Biol.

[27] Choudhary C, Weinert BT, Nishida Y, Verdin E, Mann M (2014). The growing landscape of lysine acetylation links metabolism and cell signalling. Nat Rev Mol Cell Biol.

[28] Pena RN, Noguera JL, García-Santana MJ, González E, Tejeda JF, Ros-Freixedes R (2019). Five genomic regions have a major impact on fat composition in Iberian pigs. Sci Rep.

[29] Barea R, Isabel B, Nieto R, López-Bote C, Aguilera JF (2013). Evolution of the fatty acid profile of subcutaneous back-fat adipose tissue in growing Iberian and Landrace × Large White pigs. Animal.

[30] Benítez R, Fernández A, Isabel B, Núñez Y, De Mercado E, Gómez-Izquierdo E (2018). Modulatory effects of breed, feeding status, and diet on adipogenic, lipogenic, and lipolytic gene expression in growing Iberian and Duroc pigs. Int J Mol Sci.

[31] Ventanas S, Tejeda JF, Estévez M (2008). Chemical composition and oxidative status of tissues from Iberian pigs as affected by diets: extensive feeding v. oleic acid- and tocopherol-enriched mixed diets. Animal..

[32] Chen XF, Chen X, Tang X (2020). Short-chain fatty acid, acylation and cardiovascular diseases. Clin Sci.

[33] Bolsoni-Lopes A, Alonso-Vale MIC (2015). Lipolysis and lipases in white adipose tissue - An update. Arch Endocrin Metab.

[34] Liu X, Liang Y, Song R, Yang G, Han J, Lan Y (2018). Long non-coding RNA NEAT1-modulated abnormal lipolysis via ATGL drives hepatocellular carcinoma proliferation. Mol Cancer.

[35] Zimmermann R, Strauss JG, Haemmerle G, Schoiswohl G, Birner-Gruenberger R, Riederer M (2004). Fat mobilization in adipose tissue is promoted by adipose triglyceride lipase. Science.

[36] Haemmerle G, Lass A, Zimmermann R, Gorkiewicz G, Meyer C, Rozman J (2006). Defective lipolysis and altered energy metabolism in mice lacking adipose triglyceride lipase. Science.

[37] Jeon YG, Kim YY, Lee G, Kim JB (2023). Physiological and pathological roles of lipogenesis. Nat Metab.

[38] Kim IH, Nam TJ (2017). Enzyme-treated Ecklonia cava extract inhibits adipogenesis through the downregulation of C/EBPα in 3T3-L1 adipocytes. Int J Mol Med.

[39] Jaenisch R, Young R (2008). Stem cells, the molecular circuitry of pluripotency and nuclear reprogramming. Cell.

[40] Meissner A (2010). Epigenetic modifications in pluripotent and differentiated cells. Nat Biotechnol.

[41] Li TY, Song L, Sun Y, Li J, Yi C, Lam SM (2018). Tip60-mediated lipin 1 acetylation and ER translocation determine triacylglycerol synthesis rate. Nat Commun.

[42] Shanaki M, Omidifar A, Shabani P, Toolabi K (2022). Association between HDACs and pro-inflammatory cytokine gene expressions in obesity. Arch Physiol Biochem.

[43] Feng B, Jiao P, Helou Y, Li Y, He Q, Walters MS (2014). Mitogen-activated protein kinase phosphatase 3 (MKP-3)-deficient mice are resistant to diet-induced obesity. Diabetes.

