# Supplementary figures and images for: Lysine 2-hydroxyisobutyrylation levels determined adipogenesis and fat accumulation in adipose tissue in pigs
Source: J Anim Sci Biotechnol. 2024 Jul 12;15:99. doi: 10.1186/s40104-024-01058-9 (PMC11242017; doi:10.1186/s40104-024-01058-9)

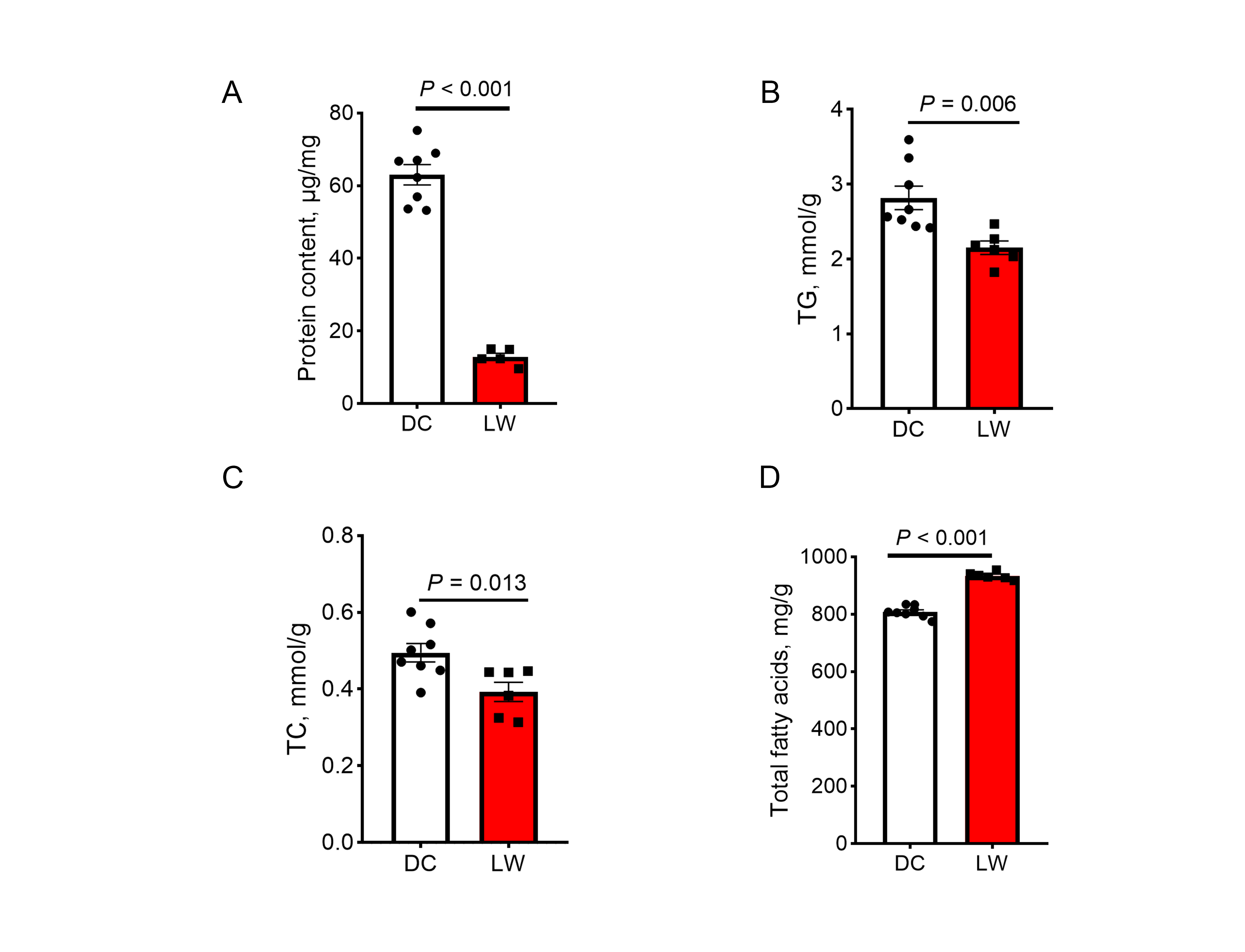

Supplement: Supplementary file 1 — Additional file 1. Fig. S1 Comparison of TC, TG, total fatty acid and protein contents of backfat between obese- and lean-type pigs. TC, total cholesterol. TG, Triglyceride. DC, Duroc pig (lean-type pig). LW, Laiwu pig (obese-type pig). The data are presented as the mean ± SEM, n = 6, 7, or 8. [file 40104_2024_1058_MOESM1_ESM.tif]

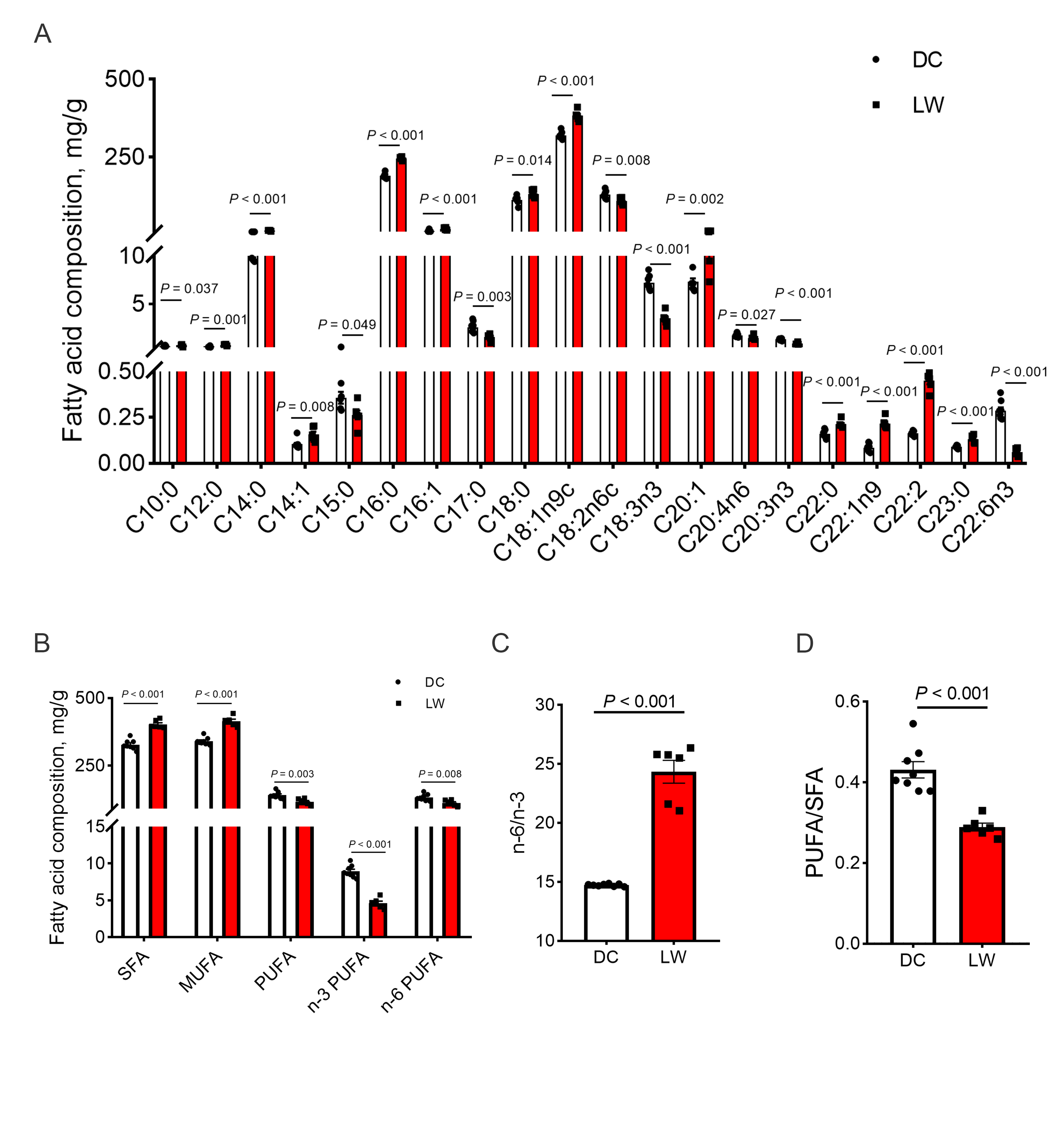

Supplement: Supplementary file 2 — Additional file 2. Fig. S2 Comparison of fatty acid composition of backfat between obese- and lean-type pigs. A and B Fatty acid composition of backfat in obese- and lean-type pigs (n = 6, 7 or 8). C The ratio of n-6/n-3 polyunsaturated fatty acids (PUFA) of backfat in obese- and lean-type pigs (n = 6, 7 or 8). D The PUFA/SFA ratio of backfat in obese- and lean-type pigs (n = 6, 7, or 8). DC, Duroc pig (lean-type pig). LW, Laiwu pig (obese-type pig). The data are presented as the mean ± SEM. [file 40104_2024_1058_MOESM2_ESM.tif]

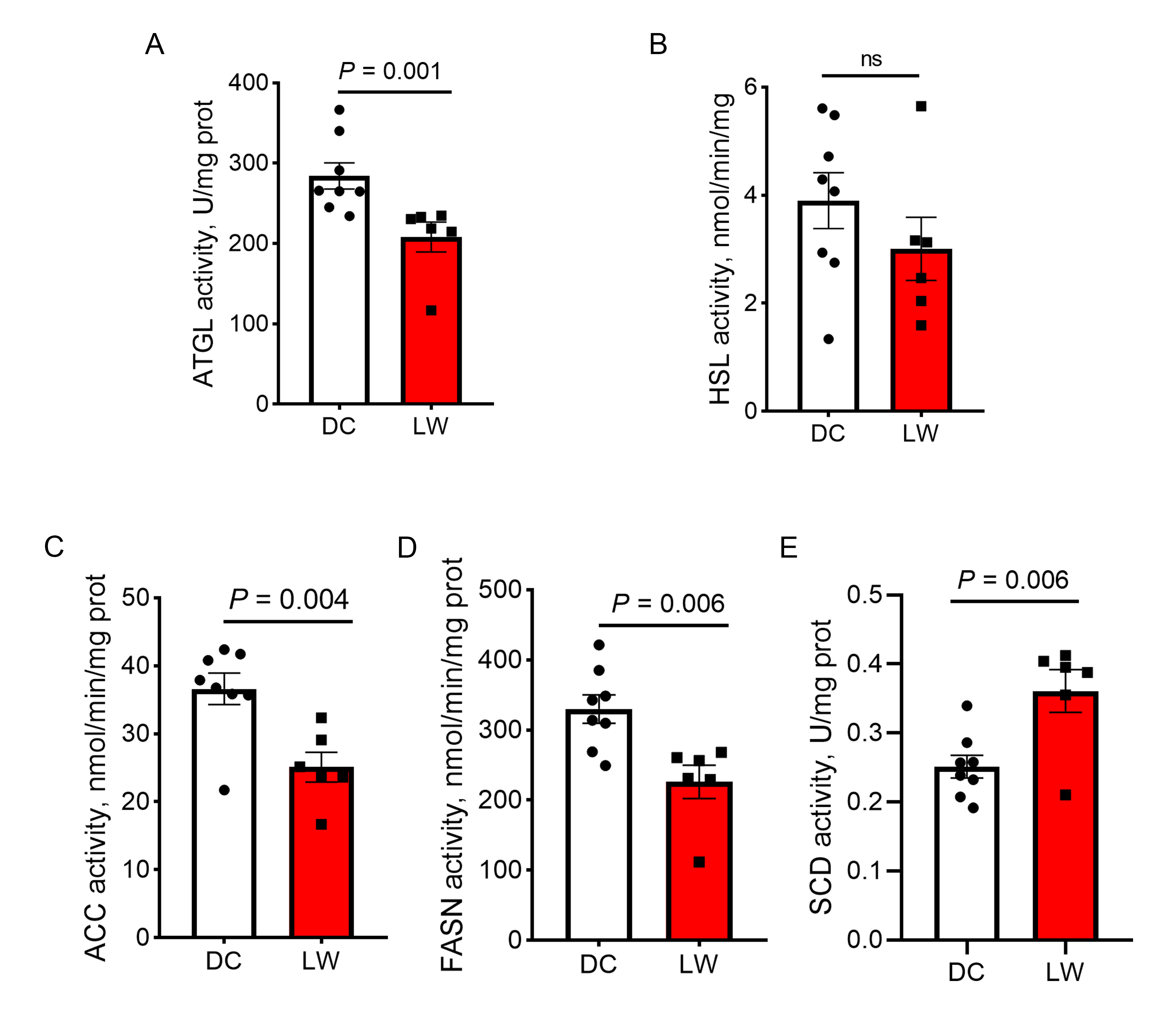

Supplement: Supplementary file 3 — Additional file 3. Fig. S3 Comparison of activities of enzymes related to fatty acid metabolism of backfat between obese- and lean-type pigs. DC, Duroc pig (lean-type pig). LW, Laiwu pig (obese-type pig). The data are presented as the mean ± SEM, n = 6, 7 or 8. [file 40104_2024_1058_MOESM3_ESM.tif]
