# Supplementary material for: Lysine 2-hydroxyisobutyrylation levels determined adipogenesis and fat accumulation in adipose tissue in pigs
Source: J Anim Sci Biotechnol. 2024 Jul 12;15:99. doi: 10.1186/s40104-024-01058-9 (PMC11242017; doi:10.1186/s40104-024-01058-9)
Supplement: Supplementary file 4 — Additional file 4. Table S1 Sequences of siRNA. Table S2 Antibodies information. Table S3 Primer sequences of target genes used for qRT-PCR assays. Table S4 Differential regulatory pathways of Khib modified proteins of FAPs and MSCs in GSEA. [file 40104_2024_1058_MOESM4_ESM.docx]

**Table S1** Sequences of siRNA

| **siRNA name** | **Sequence (5′ to 3′)** |
| --- | --- |
| siKAT5 | Sense: 5′ AGUGUUUCCUCGACCACAAGA 3′ |
|  | Anti-sense: 5′ UUGUGGUCGAGGAAACACUUG 3′ |
| siHDAC2 | Sense: 5′ CCAAUGAGUUGCCAUAAUU 3′ |
|  | Anti-sense: 5′ UUAUAUGGCAACUCAUUGGUU 3′ |
| siCON | Sense: 5′ UUCUCCGAACGUGUCACGUTT 3′ |
|  | Anti-sense: 5′ ACGUGACACGUUCGGAGAATT 3′ |

Table S2 Antibodies information

| **Antibodies** | **Source** | **Identifier** | **Dilution** |
| --- | --- | --- | --- |
| Anti-Khib | PTM BIO | Cat# PTM-802 | 1:1,000 |
| Anti-Kac | PTM BIO | Cat# PTM-105 | 1:1,000 |
| Anti-Kcr | PTM BIO | Cat# PTM-501 | 1:1,000 |
| Anti-Ksu | PTM BIO | Cat# PTM-401 | 1:1,000 |
| Anti-KAT5 | Proteintech | Cat# 10827-1-AP | 1:1,000 |
| Anti-HDAC2 | Proteintech | Cat# 12922-3-AP | 1:1,000 |
| Anti-β-actin | Proteintech | Cat# 66009-1-Ig | 1:20,000 |
| Anti-β-tubulin | Cell Signaling Technology | Cat# 2146 | 1:1,000 |
| DyLight™ 800 Anti-rabbit IgG (H + L) | Cell Signaling Technology | Cat# 5151 | 1:10,000 |
| DyLight™ 800 Anti-mouse IgG (H + L) | Cell Signaling Technology | Cat# 5257 | 1:10,000 |

**Table S3** Primer sequences of target genes used for qRT-PCR assays

| **Gene name** | **Primer sequence (5′ to 3′)** | **Tm, ℃** | **Product size, bp** |
| --- | --- | --- | --- |
| *KAT5* | F: 5′ GATCACACTCCGCTTCAACC 3′ | 60 | 112 |
|  | R: 5′ CCACCTTCCGTTTCGTTGAG 3′ |  |  |
| *HDAC2* | F: 5′ CTGCCAGTTCAACCTTCACC 3′ | 60 | 158 |
|  | R: 5′ AGCAGACTTTCTTCTTGCCG 3′ |  |  |
| *GAPDH* | F: 5′ TCGGAGTGAACGGATTTG 3′ | 60 | 219 |
|  | R: 5′ CCTGGAAGATGGTGATGG 3′ |  |  |

| **Table S4** Differential regulatory pathways of Khib modified proteins of FAPs and MSCs in GSEA | | | | |
| --- | --- | --- | --- | --- |
| **Pathway** | **ES** | **NES** | **FDR q-val** | **FWER *P*-val** |
| **Up-regulated** |  |  |  |  |
| Aminoacyl-trna Biosynthesis | 0.456 | 4.896 | ＜ 0.001 | ＜ 0.001 |
| Hypertrophic Cardiomyopathy (HCM) | 0.365 | 4.133 | ＜ 0.001 | ＜ 0.001 |
| Dilated Cardiomyopathy (DCM) | 0.359 | 4.130 | ＜ 0.001 | ＜ 0.001 |
| Progesterone-mediated Oocyte Maturation | 0.416 | 3.656 | ＜ 0.001 | ＜ 0.001 |
| Th17 Cell Differentiation | 0.396 | 3.441 | ＜ 0.001 | ＜ 0.001 |
| Adrenergic Signaling in Cardiomyocytes | 0.3380 | 3.081 | ＜ 0.001 | ＜ 0.001 |
| Cardiac Muscle Contraction | 0.337 | 3.011 | ＜ 0.001 | ＜ 0.001 |
| RNA Transport | 0.268 | 2.973 | ＜ 0.001 | ＜ 0.001 |
| Hepatocellular Carcinoma | 0.589 | 2.793 | ＜ 0.001 | ＜ 0.001 |
| Apoptosis | 0.303 | 2.778 | ＜ 0.001 | ＜ 0.001 |
| Ribosome | 0.137 | 2.760 | ＜ 0.001 | ＜ 0.001 |
| Ferroptosis | 0.589 | 2.757 | ＜ 0.001 | ＜ 0.001 |
| Nod-like Receptor Signaling Pathway | 0.274 | 2.690 | ＜ 0.001 | 0.002 |
| Porphyrin and Chlorophyll Metabolism | 0.430 | 2.501 | ＜ 0.001 | 0.007 |
| **Down-regulated** |  |  |  |  |
| Protein Processing in Endoplasmic Reticulum | -0.272 | -4.878 | ＜ 0.001 | ＜ 0.001 |
| Thyroid Hormone Synthesis | -0.466 | -4.179 | ＜ 0.001 | ＜ 0.001 |
| Huntington Disease | -0.364 | -3.749 | ＜ 0.001 | ＜ 0.001 |
| Human T-cell leukemia Virus 1 Infection | -0.344 | -3.606 | ＜ 0.001 | ＜ 0.001 |
| Tryptophan Metabolism | -0.544 | -3.362 | ＜ 0.001 | ＜ 0.001 |
| Lysine Degradation | -0.501 | -3.319 | ＜ 0.001 | ＜ 0.001 |
| Mapk Signaling Pathway | -0.274 | -3.209 | ＜ 0.001 | ＜ 0.001 |
| Parkinson Disease | -0.318 | -3.191 | ＜ 0.001 | ＜ 0.001 |
| Valine, Leucine and Isoleucine Degradation | -0.495 | -3.051 | ＜ 0.001 | ＜ 0.001 |
| Fatty Acid Degradation | -0.467 | -3.047 | ＜ 0.001 | ＜ 0.001 |
| Cholesterol Metabolism | -0.453 | -2.928 | ＜ 0.001 | ＜ 0.001 |
| Human Cytomegalovirus Infection | -0.313 | -2.884 | ＜ 0.001 | ＜ 0.001 |
| Fatty Acid Metabolism | -0.420 | -2.855 | ＜ 0.001 | ＜ 0.001 |
| Butanoate Metabolism | -0.515 | -2.840 | ＜ 0.001 | ＜ 0.001 |
| Herpes Simplex Virus 1 Infection | -0.311 | -2.825 | ＜ 0.001 | ＜ 0.001 |
| Alcoholism | -0.371 | -2.756 | ＜ 0.001 | 0.002 |
| Citrate Cycle (TCA Cycle) | -0.324 | -2.754 | ＜ 0.001 | 0.002 |
| Human Immunodeficiency Virus 1 Infection | -0.301 | -2.753 | ＜ 0.001 | 0.002 |
| Fatty Acid Elongation | -0.505 | -2.7450 | ＜ 0.001 | 0.002 |
| Calcium Signaling Pathway | -0.371 | -2.740 | ＜ 0.001 | 0.002 |
| Epstein-barr Virus Infection | -0.234 | -2.649 | ＜ 0.001 | 0.004 |
| Oxidative Phosphorylation | -0.332 | -2.600 | ＜ 0.001 | 0.006 |
| Ecm-receptor Interaction | -0.334 | -2.600 | ＜ 0.001 | 0.006 |
| Propanoate Metabolism | -0.376 | -2.553 | ＜ 0.001 | 0.007 |
| Alzheimer Disease | -0.246 | -2.523 | ＜ 0.001 | 0.011 |
| Focal Adhesion | -0.180 | -2.477 | ＜ 0.001 | 0.012 |
| Longevity Regulating Pathway - Multiple Species | -0.514 | -2.470 | ＜ 0.001 | 0.013 |
| Cellular Senescence | -0.310 | -2.412 | ＜ 0.001 | 0.026 |
| Transcriptional Misregulation in cancer | -0.550 | -2.389 | 0.001 | 0.030 |
| Systemic Lupus Erythematosus | -0.279 | -2.325 | 0.002 | 0.050 |

14 pathways were significantly up-regulated (FDR q-val < 0.05, FWER *P*-val < 0.05), 30 pathways were significantly down-regulated (FDR q-val < 0.05, FWER *P*-val < 0.05). Contrast strategy: MSCs *vs* FAPs, *n* = 3
